# Molecular Characterization, Expression, Evolutionary Selection, and Biological Activity Analysis of *CD68* Gene from *Megalobrama amblycephala*

**DOI:** 10.3390/ijms232113133

**Published:** 2022-10-28

**Authors:** Hujun Cui, Hong Li, Minying Zhang, Hongping Li, Xu Wang, Zirui Wang, Wei Zhai, Xiangning Chen, Hanliang Cheng, Jianhe Xu, Xiaoheng Zhao, Zhujin Ding

**Affiliations:** 1 Jiangsu Key Laboratory of Marine Bioresources and Environment, Co-Innovation Center of Jiangsu Marine Bio-Industry Technology, Jiangsu Ocean University, Lianyungang 222005, China; 2 Jiangsu Key Laboratory of Marine Biotechnology, School of Marine Science and Fisheries, Jiangsu Ocean University, Lianyungang 222005, China; 3 Hunan Fisheries Science Institute, Changsha 410153, China

**Keywords:** *Megalobrama amblycephala*, *CD68* gene, expression patterns, evolutionary analysis, recombinant protein, biological activities

## Abstract

CD68 is a highly glycosylated transmembrane glycoprotein that belongs to the lysosome-associated membrane glycoprotein family and is involved in various immune processes. In this study, *Megalobrama amblycephala CD68* (*MaCD68*) was cloned and characterized, and its expression patterns and evolutionary characteristics were analyzed. The coding region of *MaCD68* was 987 bp, encoding 328 amino acids, and the predicted protein molecular weight was 34.9 kDa. MaCD68 contained two transmembrane helical structures and 18 predicted N-glycosylation sites. Multiple sequence alignments showed that the MaCD68 protein had high homology with other fish, and their functional sites were also highly conserved. Phylogenetic analysis revealed that *MaCD68* and other cypriniformes fish clustered into one branch. Adaptive evolution analysis identified several positively selected sites of teleost *CD68* using site and branch-site models, indicating that it was under positive selection pressure during evolution. Quantitative real-time reverse transcription polymerase chain reaction analysis showed that *MaCD68* was highly expressed in the head kidney, spleen, and heart. After *Aeromonas hydrophila* infection, *MaCD68* was significantly upregulated in all tested tissues, peaking at 12 h post-infection (hpi) in the kidney and head kidney and at 120 hpi in the liver and spleen, suggesting that *MaCD68* participated in the innate immune response of the host against bacterial infection. Immunohistochemical and immunofluorescence analyses also showed that positive signals derived from the MaCD68 protein were further enhanced after bacterial and lipopolysaccharide treatment, which suggested that MaCD68 is involved in the immune response and could be used as a macrophage marker. Biological activity analysis indicated that recombinant MaCD68 (rMaCD68) protein had no agglutination or bactericidal effects on *A. hydrophila* but did have these effects on *Escherichia coli*. In conclusion, these results suggest that MaCD68 plays a vital role in the immune response against pathogens, which is helpful in understanding the immune responses and mechanisms of *M. amblycephala*.

## 1. Introduction

CD68 is a highly glycosylated type I transmembrane glycoprotein [1] that is structurally similar to lysosome-associated membrane proteins (LAMPs; thusly also called “LAMP-4”) and belongs to the LAMP glycoprotein family [2]. Compared to LAMP-1 and LAMP-2, CD68 contains only one LAMP-like domain, including two conserved disulfide bonds and a mucin-like domain, which resembles LAMP3 [2,3]. CD68 is involved in inflammatory immunity [4,5], cancer diagnosis and prognosis [6], and osteoclast bone resorption [7]. CD68 can differentiate M1 and M2 macrophage polarization by binding to the transcription factor markers pSTAT1, RBP-J, and CMAF [8].

CD68 is mainly localized in the endosomal/lysosomal compartments of macrophages [1] and is highly expressed in mononuclear macrophage cell lines such as macrophages, microglia, osteoclasts, and myeloid dendritic cells [9]. Low expression of CD68 is found in non-hematopoietic cells, including human arterial intimal smooth muscle cells [10], fibroblasts, endothelial cells, various tumor cell lines [11], human umbilical cord mesenchymal stem cells [12], and non-hematopoietic tissues and bodily fluids such as glomeruli, serum, and urine [13]. Additionally, expression levels of CD68 are low in CD19^+^ B lymphocytes and mature CD14^+^ monocytes [14].

Most studies on CD68 have focused on mammals, with very few reports on teleosts. The expression of CD68 in the hematopoietic tissues of *Carassius auratus* L. increases gradually with the maturation of macrophages, and the upregulation of expression levels is significant from early progenitor cells to mature macrophages [15]. Macrophages, monocytes, and their progenitor cells in the head kidney lymphocytic aggregates of *Centropomus parallelus* are strongly immunopositive for CD68 [16]. However, immunohistochemical analysis shows no immune reactions to an anti-CD68 monoclonal antibody in the spleen, kidney, and liver sections of *Salmo Gairdneri* Richardson [17]. *Larimichthys crocea CD68* (*LcCD68*) mRNA is expressed in primary macrophages and in two established macrophage cell lines (LCM07 and LCM10), all of which are derived from the head kidney [18].

*Megalobrama amblycephala* is one of the most economically important cultured fish in China. However, septicemia caused by *Aeromonas hydrophila* infection has become the main factor restricting the sustainable development of *M. amblycephala* cultures. Studies on the immune system and disease resistance mechanisms of *M. amblycephala* can provide a theoretical basis for disease control. Understanding the role and mechanism of CD68 as a typical macrophage marker in innate immunity may lay the foundation for further exploration of immune defense mechanisms in *M. amblycephala*. In this study, we cloned and characterized the *M. amblycephala CD68* (*MaCD68*) gene, analyzed the evolutionary characteristics of the *CD68* gene in fish, and detected the expression and distribution of the *MaCD68* gene and protein before and after infection. In addition, MaCD68 could be used as a macrophage marker and its recombinant protein had excellent bacterial binding, agglutination and inhibitory activities in vitro, which provided support for elucidating the immune response mechanism of *M. amblycephala* against bacterial infection.

## 2. Results

### 2.1. Sequence Analysis of MaCD68

In this study, the cDNA sequence of *MaCD68* was identified and characterized. The coding region of *MaCD68* was 987 bp and encoded 328 amino acids (aa) (GenBank accession No. OP650090). The predicted molecular weight of the encoded protein was 34.9 kDa, and the theoretical isoelectric point was 8.93. The aliphatic aa index was 70.88, and the total mean value of hydrophilicity (GRAVY) was −0.329. The total number of negatively charged (Asp + Glu) and positively charged (Arg + Lys) residues was 19 and 24, respectively.

A predicted possible MaCD68 cleavage point was between aa 22 and 23, and further analysis predicted that this was a signal peptide rather than a signal anchor. The MaCD68 aa sequence had a LAMP domain and two transmembrane helical structures, of which the first was between 5 and 27 aa, the second was between 295 and 317 aa; 28 to 294 aa comprised the extracellular region. In addition, the MaCD68 aa sequence might have 18 N-glycosylation sites with a threshold of 0.5. Sequence characteristic analysis of the *MaCD68* gene would be helpful for the multiple sequences alignment, evolutionary analysis and further biological functions analysis.

### 2.2. Multiple Sequence Alignment and Phylogenetic Analysis of MaCD68

**Figure 1:**
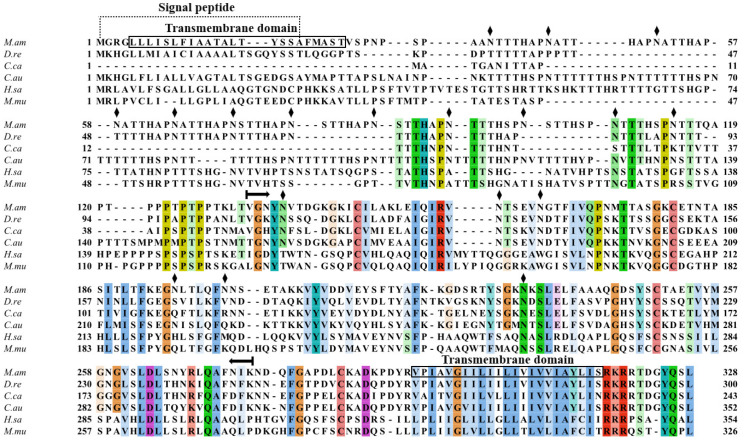
Multiple sequence alignment of CD68 from various species. *M.am*: *Megalobrama amblycephala*; *D.re*: *Danio rerio*; *C.ca*: *Cyprinus carpio*; *C.au*: *Carassius auratus*; *H.sa*: *Homo sapiens*; *M.mu*: *Mus musculus.* Residues with >50% similarity are shaded. Black rhombuses represent N-glycosylation sites. A lysosome-associated membrane protein (LAMP) domain was presented between the two horizontal arrows.

Multiple sequence alignments of MaCD68 homologues from *Danio rerio*, *Cyprinus carpio*, *Carassius auratus*, *Homo sapiens* and *Mus musculus* were performed (Figure 1). The MaCD68 protein was highly homologous to that of other fish but shared low homology with the mammalian CD68 protein. The key functional sites of *M. amblycephala* CD68 and of other aligned species were completely conserved, indicating that the biological functions of the CD68 protein have been maintained during evolution.

**Figure 2:**
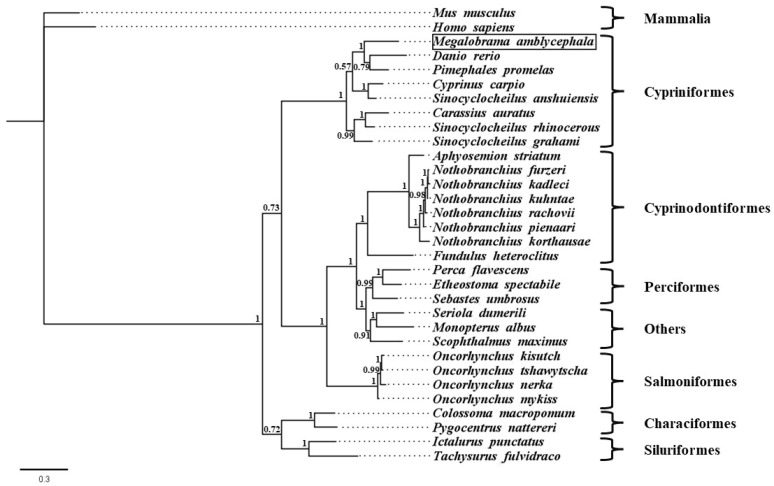
Phylogenetic analysis of teleost *CD68* genes. *M. amblycephala* was indicated by a box.

Phylogenetic analysis showed that *CD68* genes from *M. amblycephala* and other cypriniformes were clustered into one branch, while those from other fish and mammals were clustered into other branches (Figure 2). Therefore, the constructed phylogenetic tree was reliable and could be used for further adaptive evolutionary analyses.

### 2.3. Adaptive Evolutionary Analysis

Positive adaptive evolution of a gene can be inferred when the ratio of non-synonymous (*d_N_*) to synonymous (*d_S_*) substitution rates is greater than one. Adaptive evolutionary analysis of *CD68* in teleosts was performed using the site and branch-site models of PAML software. In the site model, five positively selected sites (T46, H97, K252, A265, and K305) were identified under the assumption that ω was greater than one in the M2a and M8 models (Table 1). In the branch-site model, Cypriniformes, Cypriniformes, Perciformes, Salmoniformes, Characiformes, Siluriformes, Carangiformes, Synbranchiformes, Pleuronectiformes, and Teleost fish were set as the foreground branches in rotation, and the other branches were set as the background branch. Two positive selection sites (R225 and G313) were detected in Characiformes, one (N360) in Perciformes, and one (Y297) in Teleost fish (Table 2 and Table 3). These results provide evidence for the adaptive evolution of the teleost *CD68* gene, indicating that it was under positive selection pressure during evolution.

### 2.4. Expression of MaCD68 Gene in Healthy and Infected Tissues

**Figure 3:**
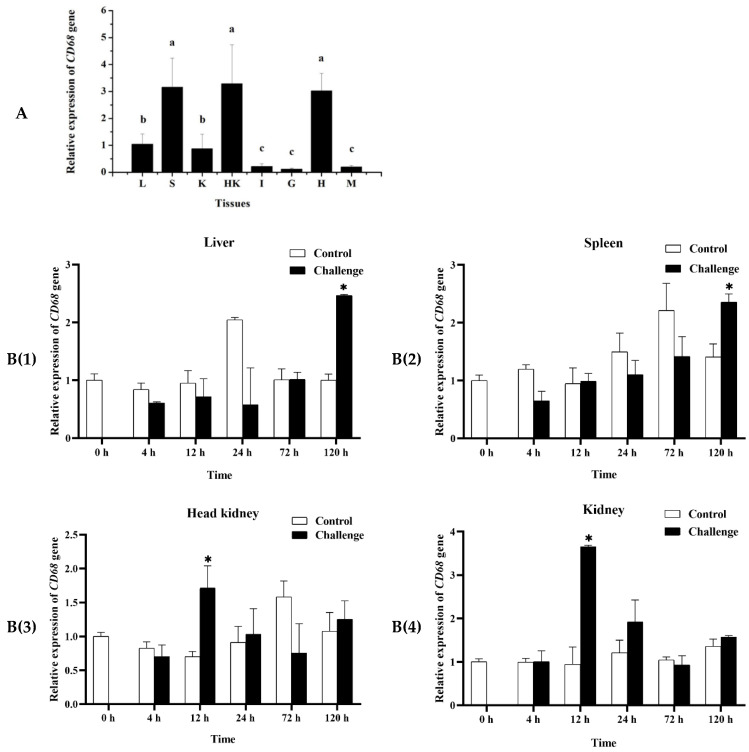
Expression patterns of *MaCD68* gene in healthy tissues (**A**) and upon bacterial infection (**B**). (**A**) L: liver, S: Spleen, K: Kidney, HK: Head Kidney, I: Intestine, G: Gill, H: Heart, M: Muscle. Different superscript letters indicate statistically significant differences (*p* < 0.05). (**B**) Asterisks indicate notable statistical differences (*p* < 0.05). **B(1)**–**B(4)** were liver, spleen, head kidney and kidney post bacterial infection, respectively.

Quantitative real-time reverse transcription polymerase chain reaction (qRT-PCR) was performed to detect the expression patterns of *MaCD68* in healthy tissues and upon infection, thereby providing a basis for further study of its biological functions in juvenile *M. amblycephala*. The results showed that *MaCD68* was expressed in all tested tissues (Figure 3A), and its expression levels in the head kidney, spleen, and heart were significantly higher than those in other tissues (*p* < 0.05), followed by the liver and body kidney, and were extremely low in the intestine, gill, and muscle tissues. As a typical macrophage marker, the tissue expression pattern of MaCD68 indicated that it is a vital component of the host immune system.

*MaCD68* expression was significantly upregulated in the kidney, head kidney, liver, and spleen of *M. amblycephala* infected with *A. hydrophila* (Figure 3B). In the kidney and head kidney, *MaCD68* reached its peak level 12 h post-infection (hpi) and then decreased gradually. *MaCD68* was not upregulated in the liver and spleen until 120 hpi. The expression patterns upon infection revealed the participation of *MaCD68* in host immune defense responses, particularly those of the kidney and head kidney tissues, and its roles in different immune-related tissues may be diverse.

### 2.5. Preparation of Recombinant Protein and Polyclonal Antibody

**Figure 4:**
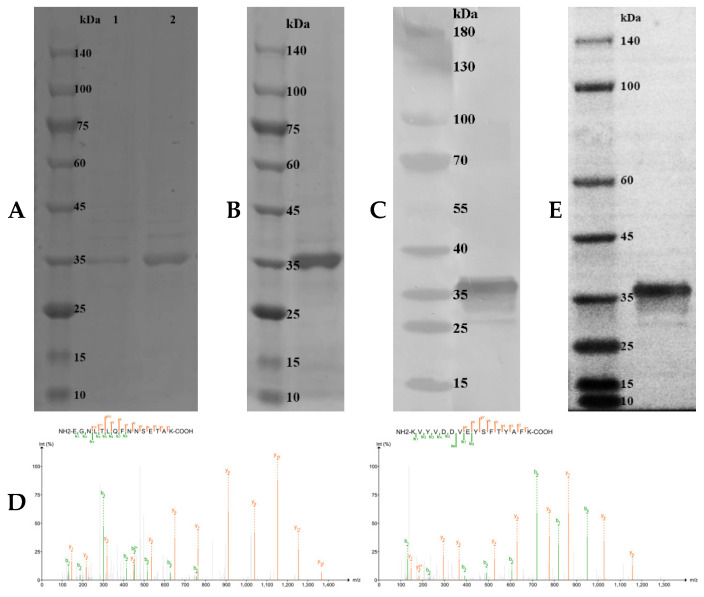
Detection of the prepared rMaCD68 protein and antibody. (**A**) SDS-PAGE analysis of the induced rMaCD68 protein. Lane 1: total proteins of uninduced *Escherichia coli* BL21 (DE3)-competent cells transformed with pET32a-MaCD68, Lane 2: total proteins of induced *E. coli* BL21 (DE3)-competent cells transformed with pET32a-MaCD68. (**B**) SDS-PAGE analysis of the purified rMaCD68 protein. (**C**) Identification of recombinant MaCD68 by Western blotting with His-tag Antibody. (**D**) Identification of purified product by Mass Spectrometry. (**E**) Specificity analysis of the prepared MaCD68 antibody by Western blotting.

To analyze biological activities, recombinant MaCD68 (rMaCD68) protein was prepared by isopropyl-β-D-thiogalactopyranoside (IPTG) induction of the pET-32a prokaryotic expression system and purified using a Ni-agarose His-tagged protein purification kit. SDS-PAGE analysis showed that the molecular weight of the induced and purified rMaCD68 protein was approximately 37 kDa, which was consistent with the calculated molecular weight, and was verified by His-tag antibody (Figure 4A–C). Moreover, the purified product was identified as rMaCD68 by mass spectrometry (Figure 4D). Western blotting revealed that the prepared polyclonal antibody against MaCD68 exhibited high specificity (Figure 4E) and could be used in further studies.

### 2.6. Biological Activities of rMaCD68 Protein

**Figure 5:**
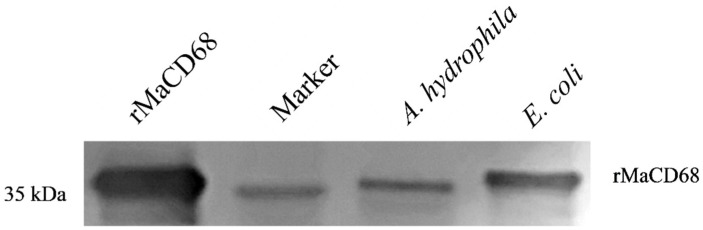
Detection of bacterial binding activity of rMaCD68 by Western blotting.

**Figure 6:**
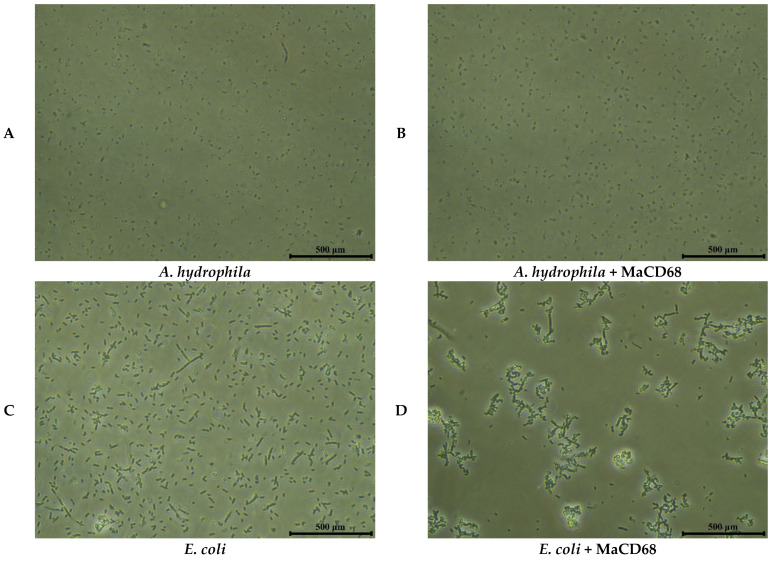
Bacterial agglutination activity of rMaCD68 protein. (**A**,**B**) *A. hydrophila*; (**C**,**D**) *E. coli*. Scale bars = 500 μm.

**Figure 7:**
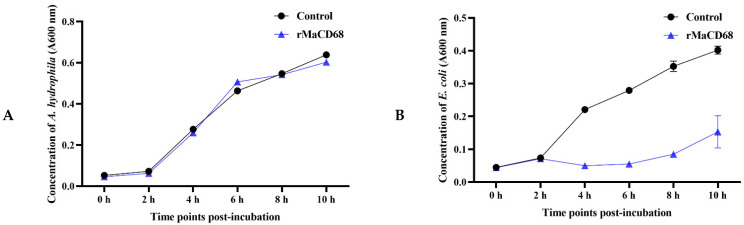
Antibacterial activity of rMaCD68 against *A. hydrophila* (**A**) and *E. coli* (**B**). The proliferation of *E. coli* and *A. hydrophila* incubated with active or inactivated rMaCD68 protein at different time points was calculated according to the optical absorbance at 600 nm. The data are presented as means ± SE (*n* = 3).

In the present study, the bacterial binding, agglutination and antibacterial activities were analyzed to evaluate the biological functions of rMaCD68 protein. The binding activity of rMaCD68 to *A. hydrophila* and *E. coli* was determined by Western blotting after the bacteria were incubated with rMaCD68 for 1 h. The results showed that rMaCD68 could bind to both *E. coli* and *A. hydrophila* (Figure 5). Furthermore, we assessed the bacterial agglutination activity of rMaCD68. As shown in Figure 6, rMaCD68 agglutinated *E. coli*, but its agglutination of *A. hydrophila* was not obvious. Additionally, the antimicrobial activities (absorbance at 600 nm) of rMaCD68 against *E. coli* and *A. hydrophila* were compared at different incubation time points. When compared with the control group, rMaCD68 did not effectively inhibit the proliferation of *A. hydrophila* but had excellent inhibitory activity against *E. coli* (Figure 7). These results revealed that the bacterial binding, agglutination and antibacterial activities of rMaCD68 protein against different bacteria were significantly different, which might be related to their membrane protein compositions.

### 2.7. Immunohistochemical Analysis of MaCD68 Protein

**Figure 8:**
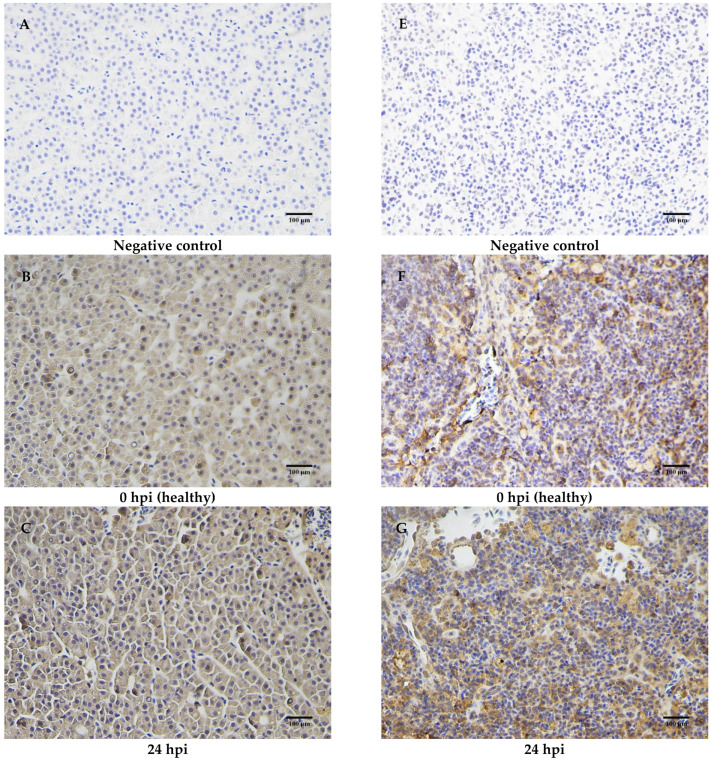

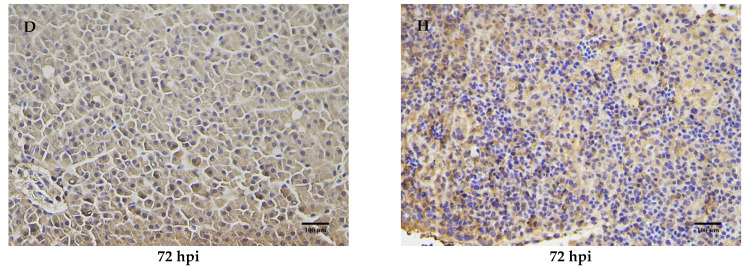
Immunohistochemical analysis of MaCD68 in healthy and *A. hydrophila*-infected *M. amblycephala* liver (**A**–**D**) and spleen (**E**–**H**) sections. (**A**,**E**) negative control; (**B**,**F**) healthy liver and spleen sections, respectively; (**C**,**D**) liver sections at 24 and 72 h post infection (hpi), respectively; (**G**,**H**) spleen sections at 24 and 72 hpi, respectively. Scale bars = 100 μm.

To determine the expression level and distribution of MaCD68 protein in healthy and infected juvenile fish, the liver and spleen were selected for immunohistochemical analysis according to the qRT-PCR results. In both the liver and spleen sections, no positive signal was found in the negative control groups that were incubated with pre-immune serum instead of the primary antibody against MaCD68 (Figure 8A,E). In healthy *M. amblycephala* liver sections, the positive immunoreactive signals for MaCD68 were mainly distributed in the cytoplasm, with a minor portion in the nucleus (Figure 8B). These parameters were significantly enhanced after bacterial infection (Figure 8C,D). In addition, strong positive immunohistochemical reactions for MaCD68 were observed in healthy spleen sections, which were further enhanced upon bacterial infection (Figure 8F–H). The increased expression of MaCD68 protein after bacterial infection indicated that MaCD68 is involved in the host immune defense response and the phagocytosis of macrophages in these tissues.

### 2.8. Subcellular Localization of MaCD68

**Figure 9:**
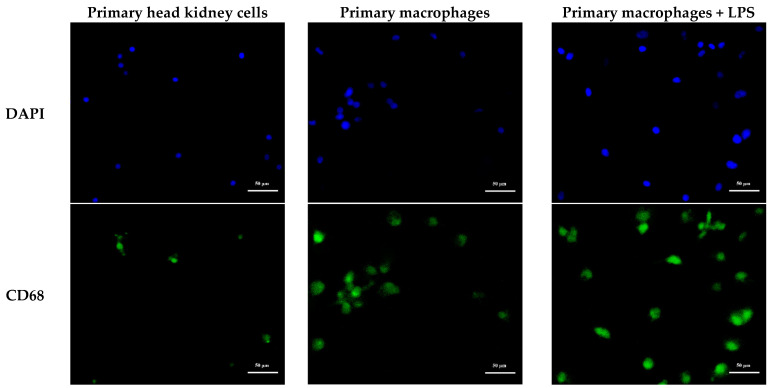

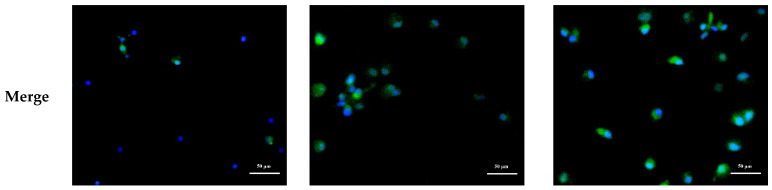
Immunofluorescence analysis of MaCD68 protein. Distribution of MaCD68 protein in the primary head kidney cells, primary macrophages, and primary macrophages treated with LPS for 4 h. Scale bar = 50 μm. Blue was DAPI staining, and green was the fluorescent second antibody binding to the first antibody specific to MaCD68.

In this study, immunofluorescence was used to detect the subcellular localization of MaCD68 in primary head kidney cells and macrophages (Figure 9). MaCD68 was expressed and distributed in only a small fraction of primary head kidney cells. In contrast, almost all primary macrophages expressed MaCD68, and positive signals were mainly found in the nucleus, and to a lesser extent in the cytoplasm. The signal intensity of MaCD68 was further enhanced in the nuclei of primary macrophages after lipopolysaccharide (LPS) treatment for 4 h and was abundantly expressed in the cytoplasm (Figure 9), revealing the upregulated expression of MaCD68 upon stimulation, which contributes to the enhanced immunity of macrophages.

## 3. Discussion

CD68 is a highly glycosylated type I transmembrane glycoprotein mainly expressed in intracellular lysosomes of monocytes and macrophages [1]. To date, CD68 has been studied mainly in mammals and rarely in lower vertebrates. The coding region of *MaCD68* consisted of a signal peptide and transmembrane domains, confirming that it is a type I transmembrane glycoprotein. N-linked glycosylation usually occurs at the Asn residue of the ASN-X-Ser/Thr motif. However, only a small number of Asn-X-Ser/Thr motifs exhibit high glycosylation efficiency, and some do not exhibit any glycosylation activity. The efficiency of N-linked glycosylation mainly depends on the type of aa after the Asn-X-Ser/Thr motif, with threonine being one of the most favorable aa [19]. There were 18 N-glycosylation sites in the MaCD68 protein, of which two-thirds of the Asn-X-Ser/Thr motif was followed by threonine, indicating that MaCD68 may be highly glycosylated. Moreover, N-glycosylation can protect CD68 from degradation during its lysosomal translocation from the Golgi network [20]. MaCD68 contained four regularly spaced cysteines (36–37 residues apart) aligned with the equivalent cysteines of *H. sapiens* CD68 and macrosialin proteins [1,2]. The LAMP family forms intra-regional disulfide bonds between the first and second cysteines and between the third and fourth cysteines [21,22]. MaCD68 had a conserved cytoplasmic tail consisting of 11 aa residues, including a conserved critical tyrosine residue preceded by glycine. In vitro mutagenesis studies have shown that this motif plays a dominant role in lysosomal targeting [3].

In the present study, adaptive evolution of the teleost *CD68* gene was detected by applying site and branch-site models using PAML software. Several positively selected sites were identified in both models, indicating that the teleost *CD68* gene has undergone adaptive evolution. However, the predicted positive selection sites did not correspond to existing functional sites, indicating that they exert no effect on the identified biological functions. Although Y297 and G313 were located in the LAMP domain, the exact function of this domain is unknown, and further studies are needed to explain their detailed functions in fish. These positively selected sites might have a certain impact on other biological functions of CD68, indicating that it has undergone adaptive evolution to acclimatize to the complex and changeable environment.

CD68 is normally used as a histochemical/cytochemical marker of inflammation-associated monocytes and macrophages [23]. As a primary lymphoid organ that is rich in monocytes and macrophages, the head kidney of teleosts plays a key role in the immune responses of fish by participating in the phagocytosis of invading pathogens and secreting cytokines [24,25,26]. Under normal physiological conditions, *MaCD68* expression was highest in the head kidney, spleen, and heart and was significantly higher than that in other healthy tissues. Similarly, transcriptome datasets indicate that *Paralichthys olivaceus CD68* is highly expressed in the heart and stomach [27]. The expression of *LcCD68* is highest in the liver, followed by the spleen [28]. In addition, *LcCD68* mRNA is highly expressed in primary macrophages and in LCM07 and LCM10 macrophage cell lines, which are all derived from the head kidney tissue [18]. These broad expression patterns suggest that CD68 has broad immune functions.

As the second primary lymphoid organ after the thymus, the head kidney produces erythrocytes, lymphocytes and granulocytes without antigen stimulation, which plays an important role in immunity [29]. The spleen is a secondary lymphoid organ that plays a relatively secondary role in the humoral immune response compared to the head kidney. After stimulation by external antigens, the spleen acts in coordination with other immune organs to activate the immune response in the body [30]. The liver also exerts certain effects on immunity. Therefore, in the present study, *MaCD68* expression in the head kidney and kidney was significantly upregulated in the early stage of acute infection (12 hpi), whereas in the spleen and liver it was significantly upregulated at 120 hpi. The significant expression levels of *MaCD68* in all tissues indicated that it is involved in antibacterial immune processes in *M. amblycephala*. The *Myxocyprinus asiaticus CD68* gene is significantly upregulated at 4 hpi with *A. hydrophila* [31], suggesting that it plays an important role in antibacterial activity. However, the expression level of the *LcCD68* gene in the liver stimulated by *Vibrio alginolyticus* is low, indicating that it plays little role in immune responses and regulation. This is presumed to be caused by the absence of certain immune domains or motifs in *LcCD68* [28]. In addition, the expression of CD68 is significantly upregulated by the inflammatory response induced by a high-fat diet. CD68 expression in the adipose tissue of *L. crocea* is significantly upregulated by an isonitrogenous diet containing a gradient of vegetable oil, suggesting that CD68 is involved in the host inflammatory response [32]. Similarly, when *M. amblycephala* are fed a high-fat diet for 60 days, the expression of MaCD68 protein in the liver is significantly increased [33]. These results reveal that teleost CD68 plays a vital role in host immune defense processes. Nevertheless, the immune function of murine CD68 differs from that of teleosts. Macrophages from CD68-deficient mice exhibit normal phagocytic activity against bacteria, and dendritic cells knocked-out for CD68 also exhibit the ability to express antigens and induce humoral immune responses, demonstrating that CD68 is not indispensable for innate and humoral immunity in mice [34].

CD68 is preferentially expressed on resident macrophages in multiple tissues, such as microglia [35], Kupffer cells [36], and osteoclasts [37], and is therefore widely used as a marker of macrophages whose expression can indicate the activity levels of macrophages [38]. Rabbit fish macrophages, a new cell line isolated and characterized from the head kidney of *Siganus fuscescens*, also show strong immunostaining signals for CD68 [39]. Consistently, significant expression of MaCD68 in *M. amblycephala* macrophages was also detected, which confirmed that fish CD68 could be used as a macrophage marker. MaCD68-positive cells were detected in the liver and spleen of healthy *M. amblycephala*, and the immunoreactivity of MaCD68 was enhanced in these tissues after *A. hydrophila* infection, which was further enhanced in macrophages because of LPS stimulation. Similarly, microglia of *Oreochromis niloticus* can be activated by LPS, leading to presentation of CD68-positive cells [40,41]. The expression of *CD68* mRNA in *L. crocea* head kidney macrophages significantly increased after treatment with recombinant *L. crocea* IFN-γ (rLcIFN-γ). Moreover, rLcIFN-γ and LPS stimulation significantly increase the CD68^+^ population of macrophages [42], and similar results are found in *P. olivaceus* head kidney macrophages [27]. In addition, double immunofluorescence staining of macrophages showed the co-localization of CD68 with IL-33, which has been recently identified as a novel biomarker and future therapeutic target for immunological disorders [43,44]. As a typical macrophage marker, the upregulated expression of CD68 upon stimulation indicates its participation in host immune responses; macrophages undergo M1 phenotypic polarization.

In this study, rMaCD68 was used to further explore its physiological functions. rMaCD68 could bind to both *A. hydrophila* and *E. coli* and was speculated to play a role in macrophage binding and phagocytosis of bacteria. However, rMaCD68 only agglutinated and inhibited the proliferation of *E. coli* but had no effect on *A. hydrophila*, which was similar to our previous results [45,46,47]. *M. amblycephala* intelectin and CD209 have no agglutination or bactericidal effects on *A. hydrophila* but have these effects on *E. coli*. This phenomenon has also been reported in other studies, such as *Plecoglossus altivelis* CD302 [48] and common carp intelectin [49] binding to *A. hydrophila* rather than agglutination. It should be CD68 that recognizes *A. hydrophila* in a binding manner and then initiates immune defense, rather than agglutinating them. At present, there are few studies on CD68 in fish, and the specific mechanisms involved remain unclear; therefore, further research is required.

## 4. Materials and Methods

### 4.1. Ethics Statement

This study was approved by the Animal Care and Use Committee of Jiangsu Ocean University (protocol no. 2020-37; approval date: 1 September 2019). All procedures involving animals were performed in accordance with guidelines for the Care and Use of Laboratory Animals in China.

### 4.2. Experimental Fish and Samples Collection

*M. amblycephala* was obtained from the Lianyungang Aquaculture Market. The fish were maintained in a recirculating freshwater system for 1 week. Six healthy adult fish (400 ± 30 g) were anesthetized with 3-aminobenzoic acid ethyl ester methanesulfonate (MS-222), and blood was extracted using a sterile syringe. Eight tissues, including the liver, spleen, body kidney, head kidney, intestine, gills, heart, and muscle, were collected, and 200 µL of TRIzol was added. Finally, the samples were stored in liquid nitrogen at −196 °C prior to RNA extraction.

Bacterial challenge followed a previously described method [45]. Healthy juvenile fish (14.6 ± 0.6 g) were randomly divided into challenge and control groups and were injected intraperitoneally with 0.1 mL *A. hydrophila* (1 × 10^7^ CFU/mL, half lethal dosage) and 0.6% normal saline, respectively. Thirty randomly selected individuals (divided into three pools) from each group were dissected at 0, 4, 12, 24, 72, and 120 hpi to obtain the liver, spleen, kidney, and head kidney tissues. The experimental fish were anesthetized with 100 mg/L MS-222 before dissection. After collection, the samples were frozen rapidly in liquid nitrogen and stored at −80 °C.

### 4.3. cDNA Cloning of CD68

TRIzol reagent (Cwbiotech, Beijing, China) was used to extract total RNA from all collected tissues according to the manufacturer’s instructions. The concentration of the extracted RNA was determined using a NanoDrop2000 instrument, and the RNA quality was assessed by agarose electrophoresis. First-strand cDNA was synthesized using the PrimeScript^®^ RT Reagent Kit with gDNA Eraser (TaKaRa, Dalian, China) following the manufacturer’s instructions. The *M. amblycephala* genomic region coding for *CD68* was identified through DNA database searches [50] and amplified and verified by PCR using the primers listed in Table 4. After detection on 1% agarose gels, the amplified products were ligated into the pGEM^®^-T Easy Vector (Promega, Madison, WI, USA) and sequenced by MAP Biotech (Shanghai, China). The full-length cDNA sequence was assembled using the DNAStar 7.1 software.

### 4.4. Bioinformatics Analysis

The aa composition, molecular weight, and isoelectric point of the protein-coding region of *MaCD68* were analyzed using the ExPASy website (https://web.expasy.org/protparam/ accessed on 27 March 2022). NetNGlyc 1.0, Server (https://services.healthtech.dtu.dk/service.php?NetNGlyc-1.0 accessed on 27 March 2022), was used to infer the N-glycosylation sites of the proteins. The signal peptide was predicted using SignaIP3 (http://www.cbs.dtu.dk/services/SignalP/ accessed on 27 March 2022). TMHMM (http://www.cbs.dtu.dk/services/TMHMM-2.0/ accessed on 27 March 2022) was used to analyze the transmembrane structure of the protein-coding region of the *MaCD68* gene. The protein domains were predicted using the SMART program (http://smart.embl-heidelberg.de/ accessed on 27 March 2022). The *MaCD68* gene was translated into aa using Jalview software, and the aa sequences of *M. amblycephala*, *D. rerio*, *C. carpio*, *C. auratus*, *H. sapiens* and *M. musculus* were compared and analyzed.

### 4.5. Adaptive Evolution Analysis

Thirty-two available *CD68* gene sequences (30 fish, a mouse, and a primate) were obtained from GenBank for phylogenetic analysis (Supplementary Table S1). Nucleotide sequence alignment was performed with default parameters of MUSCLE using MEGA 5.10 software [51]. The best selection strategy (GTR + I + G) was determined using jModelTest 2.1.3 software to estimate the best-fit model of *CD68* gene substitution [52]. Maximum likelihood (ML) analysis was initiated from a BIONJ tree starting with the SPR topological structure, with support values for the nodes estimated by 1000 bootstrap replicates using PhyML 3.0 [53]. Bayesian inference (BI) analysis was performed using MrBayes 3.2.1 [54], and four Markov chains were implemented for 5,000,000 generations, with the first 25% of the trees of the aging samples discarded iteratively. Markov Chain Monte Carlo runs were repeated thrice to avoid spurious results.

Selective evolution was assessed by comparing the non-synonymous/synonymous substitution ratios (*ω* = *d_N_/d_S_*), and values of *ω* (<1, =1, and >1) indicated (negative) purifying selection, neutral evolution, and positive selection, respectively [55]. The topology of the ML tree was used as a user-guide tree for positive selection analysis, which was obtained by phylogenetic analysis of the *CD68* genes. The site and branch-site models in the CODEML program of PAML 4.7 software were used to analyze the selection pressure on teleost *CD68* genes [56]. In the site model, Bayes empirical Bayes was used to calculate the Bayesian posterior probability of positive selection sites in the M2 and M8 models [57], and the statistics of each group were calculated as 2∆InL. The Chi-squared distribution-based likelihood ratio test was applied to infer a statistically significant basis for positive selection. Subsequently, the branch-site model was implemented by referring to Model A to detect positive selection among pre-specified branches (foreground) (Supplementary Table S2) [58].

### 4.6. qRT-PCR Assay

qRT-PCR was performed to analyze the expression patterns of *MaCD68* in various tissues before and after *A. hydrophila* infection, as described in our previous study [46]. Briefly, qRT-PCR was performed on a real-time PCR detection system (Bio-Rad, Berkeley, CA, USA) using the SYBR^®^ premix ExTaqTM kit (TaKaRa) in triplicate for each sample. Expression data were obtained as threshold cycle (C_t_) values, and relative gene expression levels were calculated using the 2^−∆∆Ct^ method [59] with *GAPDH* as an internal reference. The primers used are listed in Table 1. The specificity of qRT-PCR was assessed using melt curve analysis, and all reactions were performed in triplicates. The expression levels of the control group were set as “1,”; we compared control and other groups, and obtained data in the form of fold-changes.

### 4.7. Preparation of Recombinant Protein and Polyclonal Antibody

After amplification, the coding region of *MaCD68* was digested and inserted into the pET32a vector to construct a recombinant plasmid that was transformed into *E. coli* BL21 (DE3)-competent cells (TransGen Biotech, Beijing, China) using the primers listed in Table 1. The recombinant MaCD68 (rMaCD68) protein induced by IPTG at a final concentration of 0.5 mM was purified using a Ni-Agarose His-tagged Protein Purification Kit. The purified rMaCD68 protein was verified by mass spectrometry analysis and the concentration was detected using a BCA Protein Assay Kit (CoWin Biosciences, Taizhou, Jiangsu, China).

Anti-MaCD68 polyclonal antibody was prepared by subcutaneously immunizing (thrice) two Japanese white rabbits with 500 μg of recombinant MaCD68. The first injection was a mixture of recombinant protein and complete Freund’s adjuvant (Bio Basic, Inc., Toronto, ON, Canada). After an interval of 2 weeks, secondary immunizations were performed with rMaCD68 mixed with incomplete Freund’s adjuvant. Antisera were collected 10 days after the last immunization. Additionally, the antiserum was purified using a protein A/G assay, and the specificity of the antibody was tested by Western blotting (see below). Normal rabbit serum collected before the primary injection was used as a negative control.

### 4.8. Western Blotting

Western blotting was performed as previously described [45]. Protein samples in each lane were separated on a 12% SDS-PAGE gel and transferred onto polyvinylidene difluoride (PVDF) membranes using a Trans-Blot apparatus (Bio-Rad) for 2 h at 80 V. Non-specific reactivity was blocked with 5% (*w*/*v*) skim milk powder in TBS (150 mM NaCl, 20 mM Tris-base, pH 7.4) for 1 h at room temperature. The PVDF membrane was incubated with anti-MaCD68 polyclonal antibody (1:3000) overnight at 4 °C, washed thrice, and incubated with HRP-conjugated goat anti-rabbit IgG (H + L) (Beyotime, Shanghai, China; 1:2000 dilution) for 1 h at room temperature. Finally, the DAB Horseradish Peroxidase Color Development Kit (Beyotime) was used for detection.

### 4.9. Bacterial Agglutination Assay

rMaCD68 (final concentration 50 µg/mL) was mixed with *A. hydrophila* and *E. coli* suspensions (OD_600_ = 0.5) in TBS to detect the bacterial agglutination activity of the rMaCD68 protein. The mixture was vibrated at room temperature for 2 h and statically incubated for 1 h. After incubation, the suspension was observed under an inverted microscope (Nikon, Tokyo, Japan) and photographed to analyze the bacterial agglutination activity of rMaCD68 protein.

### 4.10. Bacteriostatic Activity of rMaCD68

The effect of rMaCD68 on bacterial proliferation was analyzed by measuring the bacterial density at different time points after incubation with *A. hydrophila* and *E. coli*. Bacteria cultured in LB medium to exponential phase were diluted with fresh medium at a ratio of 1:100; subsequently, rMaCD68 protein was added at a final concentration of 32 ng/μL, and inactivated rMaCD68 was used as a control. After incubation for 0, 2, 4, 6, 8, and 10 h, the optical absorbance of the mixture was measured at 600 nm to detect rMaCD68 antibacterial activity. All experiments were conducted in triplicates.

### 4.11. Bacterial Binding Assay

*E. coli* and *A. hydrophila* were fixed with formaldehyde solution and then mixed with rMaCD68 in TBS for 30 min at room temperature with continuous rotation. The bound rMaCD68 protein was examined by Western blotting with anti-MaCD68 primary antibody (1:3000) overnight at 4 °C, followed by incubation with secondary goat anti-rabbit IgG-HRP antibody (1:2000) for 1 h at room temperature, and we used a DAB kit, finally.

### 4.12. Immunohistochemistry Assay

Samples were extracted 24 and 72 h after the injection of *A. hydrophila*, and the expression and distribution of MaCD68 in the liver and spleen of the challenge and control groups were detected by immunohistochemistry (performed as previously described [45]). Fresh liver and spleen tissues were fixed with 4% paraformaldehyde at room temperature. After dehydration, transparency, and penetration, samples were embedded in paraffin. Four micrometer-thick paraffin sections were floated on water at 40 °C and dried at 37 °C. After sections were deparaffinized in xylene and rehydrated in a graded alcohol series, endogenous peroxidase was removed by immersion in 3% hydrogen peroxide. Sections were incubated with anti-MaCD68 primary antibody overnight at 4 °C and then with goat anti-rabbit IgG–HRP secondary antibody at 37 °C for 30 min. The immunoreaction products were visualized using a DAB kit, and sections were counterstained with hematoxylin, embedded in glycerol, and then observed under a light microscope (Zeiss, Oberkochen, Germany).

### 4.13. Isolation of Macrophages

Macrophages were isolated from the head kidney and kidney of *M. amblycephala* as previously described [46]. After the blood was removed, the head kidney and kidney were aseptically removed and placed in L-15 medium containing 100 U/mL penicillin, 100 μg/mL streptomycin, 100 μg/mL gentamicin, and 250 μg/mL amphotericin B in an ice bath for 30 min. A cell suspension was obtained by grinding the tissue using a 100-mesh sieve with a syringe rubber. The cell suspension was placed on top of the 31% and 45% Percoll solutions and centrifuged at 400× *g* for 30 min at 4 °C. Macrophages between 31% and 45% Percoll solution were collected, suspended in 10 mL of L-15 medium, and centrifuged at 400× *g* for 10 min at 4 °C. Finally, the precipitate was resuspended in L-15 medium containing 10% fetal bovine serum, 100 U/mL penicillin, and 0.1 mg/mL streptomycin.

### 4.14. Immunofluorescence Assay

The cell suspension and purified macrophages were inoculated into 24-well plates (500 μL/well) with round coverslips. The cells were incubated until immunofluorescence assays were performed and then washed twice with PBS. After incubation with 100 ng/μL LPS for 4 h, the cells were fixed in 400 μL 4% paraformaldehyde for 10 min at room temperature, washed twice with PBS, and permeabilized with 0.2% Triton X-100 at room temperature for 10 min. After washing, the samples were blocked with normal goat serum for 30 min; subsequently, the goat serum was removed. Macrophages were incubated with diluted anti-MaCD68 polyclonal antibody (1:2000) at 4 °C overnight and then washed with PBS thrice for 5 min each. Alexa Fluor 488-labeled goat anti-rabbit IgG (H + L) (1:500) (Beyotime) was added and incubated at room temperature for 30 min. The nuclei were stained with DAPI for 2 min and washed with PBS. Finally, the round coverslips were fixed with antifade mounting medium (Beyotime), dried at room temperature for 30 min, and observed and photographed under a fluorescence microscope (Olympus, Tokyo, Japan).

### 4.15. Statistical Analysis

In the present study, all data are presented as means ± SE. Statistical significance was assessed by one-way analysis of variance (ANOVA) using SPSS 17.0. *p*-values < 0.05 were considered statistically significant while *p*-values < 0.01 as extremely significant.

## 5. Conclusions

Herein, we cloned and characterized the *MaCD68* gene and confirmed that the teleost *CD68* gene has undergone adaptive evolution. The mRNA and protein expression levels of *MaCD68* were significantly upregulated after *A. hydrophila* infection and were involved in antibacterial immune responses. Furthermore, MaCD68 was validated as a macrophage marker. In addition, this study found that rMaCD68 exhibits excellent bacterial binding, agglutination, and inhibitory activities, which may contribute to the phagocytosis of bacteria by macrophages. These results provide an improved theoretical foundation for understanding the evolution and function of MaCD68 in various species.

## Figures and Tables

**Table 1:** Detection of positively selective sites of the teleost *CD68* gene using site model.

Gene Name	LRT *M1a* versus *M2a* (Significance)	LRT *M7* versus *M8* (Significance)	*d_N_/d_S_* (Model)	Sites ^a^ under Positive Selection
*CD68*	27.44 (*p* < 0.01)	42.02 (*p* < 0.01)	2.21 (M2a), 1.50 (M8)	T46, H97, K252, A265, K305

^a^ Results show only positively selected sites with a posterior probability (*Pb*) of ≥95%.

**Table 2:** Detection of positively selected sites of the teleost *CD68* gene using branch-site model.

Foreground Branch(es)	LRT *M1a* versus *MA* (Significance)	LRT *MA1* versus *MA* (Significance)	Sites ^a^ under Positive Selection
Characiformes	28.19 (*p* < 0.01)	23.20 (*p* < 0.01)	R225, G313
Perciformes	10.65 (*p* < 0.01)	10.65 (*p* < 0.01)	N360
Teleosts fish	15.71 (*p* < 0.01)	0 (*p* > 0.05)	Y297

^a^ Results show only positively selected sites with a posterior probability (*Pb*) of ≥95%.

**Table 3:** MA parameter results of branch-site model.

Foreground Branch(es)	Category	First Category of Sites (0)	Second Category of Sites (1)	Third Category of Sites (2a)	Fourth Category of Sites (2b)
Characiformes	Proportion	0.42459	0.46201	0.05431	0.05909
	Foreground ω	0.19077	1	998.84237	998.84237
Perciformes	Proportion	0.45428	0.50616	0.01871	0.02084
	Foreground ω	0.19187	1	999	999
Teleosts fish	Proportion	0.27506	0.33848	0.17326	0.2132
	Foreground ω	0.17821	1	1	1

**Table 4:** PCR primers used in the present study.

Names	Sequence (5′-3′)	Purpose
*CD68*-CDS-F	ATGGGACGCGGATTATTATTGATC	CDS amplification
*CD68*-CDS-R	CTATAGTGACTGGTACCCATCAG	
q*CD68*-F	CTATAGTGACTGGTACCCAT	qRT-PCR
q*CD68*-R	TGGGGAACGGTGTGAGTCTA	
q*β-actin*-F	GCTCTTACAGGAAACGGGTC	qRT-PCR
q*β-actin*-R	GCAGCAGCTCTGTAGGTCAT	
q*GAPDH*-F	TGCCGGCATCTCCCTCAA	qRT-PCR
q*GAPDH*-R	TCAGCAACACGGTGGCTGTAG	

## Supplementary Materials

The following supporting information can be downloaded at: https://www.mdpi.com/article/10.3390/ijms232113133/s1. Reference [58] is cited in the Supplementary Materials.
