# Supplementary material for: Molecular Characterization, Expression, Evolutionary Selection, and Biological Activity Analysis of CD68 Gene from Megalobrama amblycephala
Source: Int J Mol Sci. 2022 Oct 28;23(21):13133. doi: 10.3390/ijms232113133 (PMC9656401; doi:10.3390/ijms232113133)
Supplement: Supplementary file 1 [file ijms-23-13133-s001.zip › ijms-1862541-supplementary.pdf]

Supplemental Table S1. *CD68* Sequences used for phylogenetic analysis.

Species Names	Order	Family	Accession Number
Megalobrama amblycephala	Cypriniformes	Cyprinidae	KY798319.1
Danio rerio	Cypriniformes	Cyprinidae	XM_021471872.1
Pimephales promelas	Cypriniformes	Cyprinidae	XM_039659499.1
Carassius auratus	Cypriniformes	Cyprinidae	XM_026216673.1
Cyprinus carpio	Cypriniformes	Cyprinidae	XM_019094400.1
Sinocyclocheilus anshuiensis	Cypriniformes	Cyprinidae	XM_016479579.1
Sinocyclocheilus grahami	Cypriniformes	Cyprinidae	XM_016229627.1
Sinocyclocheilus rhinocerous	Cypriniformes	Cyprinidae	XM_016543127.1
Fundulus heteroclitus	Cyprinodontiformes	Fundulidae	XM_036146112.1
Aphyosemion striatum	Cyprinodontiformes	Aplocheilidae	HADW01006630.1
Nothobranchius furzeri	Cyprinodontiformes	Aplocheilidae	HAEJ01012303.1
Nothobranchius kadleci	Cyprinodontiformes	Aplocheilidae	HADZ01001372.1
Nothobranchius korthausae	Cyprinodontiformes	Aplocheilidae	HAEB01016763.1
Nothobranchius kuhntae	Cyprinodontiformes	Aplocheilidae	HAED01005512.1
Nothobranchius pienaari	Cyprinodontiformes	Aplocheilidae	HAEF01004587.1
Nothobranchius rachovii	Cyprinodontiformes	Aplocheilidae	HAEH01017065.1
Perca flavescens	Perciformes	Percidae	XM_028564140.1
Etheostoma spectabile	Perciformes	Percidae	XM_032544753.1
Sebastes umbrosus	Perciformes	Sebastidae	XM_037758134.1
Oncorhynchus kisutch	Salmoniformes	Salmonidae	XM_020491718.2
Oncorhynchus mykiss	Salmoniformes	Salmonidae	XM_021578316.2
Oncorhynchus nerka	Salmoniformes	Salmonidae	XM_029646163.1
Oncorhynchus tshawytscha	Salmoniformes	Salmonidae	XM_024405126.1
Pygocentrus nattereri	Characiformes	Serrasalmidae	XM_017718831.2
Colossoma macropomum	Characiformes	Serrasalmidae	XM_036596639.1
Ictalurus punctatus	Siluriformes	Ictaluridae	XM_017472374.1
Tachysurus fulvidraco	Siluriformes	Bagridae	XM_027169456.1
Seriola dumerili	Carangiformes	Carangidae	XM_022747801.1
Monopterus albus	Synbranchiformes	Synbranchidae	XM_020594973.1
Scophthalmus maximus	Pleuronectiformes	Scophthalmidae	XM_035623117.1
Homo sapiens	Primates	Hominidae	NM_001040059.2
Mus musculus	Rodentia	Eumuroida	NM_001291058.1

Supplemental Table S2. Branch-site model A [58].

Site Class	Proportion	Background ω	Foreground ω
0	p0	$0 < \omega0 < 1$	$0 < \omega0 < 1$
1	p1	$\omega1 = 1$	$\omega1 = 1$
2a	(1- p0- p1) p0/(p0+ p1)	$0 < \omega0 < 1$	$\omega2 \geq 1$
2b	(1- p0- p1) p1/(p0+ p1)	$\omega1 = 1$	$\omega2 \geq 1$
